# Role of P-Glycoprotein Inhibitors in the Bioavailability Enhancement of Solid Dispersion of Darunavir

**DOI:** 10.1155/2017/8274927

**Published:** 2017-10-31

**Authors:** Saleha Rehman, Bushra Nabi, Mohammad Fazil, Saba Khan, Naimat Kalim Bari, Romi Singh, Shavej Ahmad, Varinder Kumar, Sanjula Baboota, Javed Ali

**Affiliations:** ^1^Department of Pharmaceutics, School of Pharmaceutical Education & Research, Jamia Hamdard, New Delhi 110062, India; ^2^Institute of Nanoscience and Technology, Phase X, Mohali, Chandigarh, Punjab, India; ^3^Research and Development Centre, Sun Pharmaceuticals Industries Ltd., Gurgaon, Haryana, India

## Abstract

**Objective:**

The aim of the present study was to improve bioavailability of an important antiretroviral drug, Darunavir (DRV), which has low water solubility and poor intestinal absorption through solid dispersion (SD) approach incorporating polymer with P-glycoprotein inhibitory potential.

**Methods:**

A statistical approach where design of experiment (DoE) was used to prepare SD of DRV with incorporation of P-glycoprotein inhibitors. Using DoE, different methods of preparation, like melt, solvent evaporation, and spray drying method, utilizing carriers like Kolliphor TPGS and Soluplus were evaluated. The optimized SD was characterized by DSC, FTIR, XRD, and SEM and further evaluated for enhancement in absorption using everted gut sac model, effect of food on absorption of DRV, and* in vivo* prospect.

**Results and Discussion:**

DSC, FTIR, XRD, and SEM confirmed the amorphicity of drug in SD. Oral bioavailability studies revealed better absorption of DRV when given with food. Absorption studies and* in vivo* study findings demonstrated great potential of Kolliphor TPGS as P-glycoprotein inhibitor for increasing intestinal absorption and thus bioavailability of DRV.

**Conclusion:**

It is concluded that SD of DRV with the incorporation of Kolliphor TPGS was potential and promising approach in increasing bioavailability of DRV as well as minimizing its extrusion via P-glycoprotein efflux transporters.

## 1. Introduction

Acquired Immunodeficiency Syndrome (AIDS) has been one of the most devastating pandemic diseases over the last few decades caused by its etiologic agent Human Immunodeficiency Virus (HIV). Latest reports reveal that globally 40 million people are infected with HIV including 2.1 million from India in 2013 [1]. HIV is responsible for killing helper T-lymphocytes (CD4^+^ T-cells) which play a key role in the process of gaining immunity to specific pathogens, including HIV itself. No complete cure is possible for people with AIDS and life-long treatment with a combination of antiretroviral drugs; that is, Highly Active Antiretroviral Therapy (HAART) is the only therapeutic intervention with proven efficacy against HIV infection [2, 3].

HIV protease inhibitors (PIs) currently are the key components of first-line therapy in both treatment-resistant and treatment-experienced patients. The introduction of novel second-generation PIs such as Darunavir Ethanolate (DRV) with activity against wild type HIV-1 virus and multidrug resistant strains requires at least four concomitant mutations in the viral genome for resistance development, thus providing clinicians with superior drugs to counter the development of resistance [4]. DRV is coadministered with food and low dose Ritonavir (RTV), a potent CYP3A4 inhibitor as a pharmacokinetic booster to result in clinically relevant increase in the systemic exposure (bioavailability increase by up to 40%) of DRV [5, 6]. However, DRV suffer from disadvantages such as low solubility in water (0.15 mg/ml) and poor intestinal uptake due to drug efflux through active efflux transporter P-glycoprotein (P-gp) and by drug metabolism via Cytochrome P450 (CYP) 3A [7, 8].

The current clinical antiretroviral therapies have suboptimal therapeutic effect attributed to poor bioavailability of anti-HIV drugs which is due to either their poor solubility, extreme first pass metabolism, extrusion into intestine lumen by efflux transporters, drug metabolization by enzymes, or poor permeability. Therefore, there is a need for a delivery system to overcome such solubility and bioavailability issues [9]. Solid Dispersion (SD) is considered as one of the most promising strategies to enhance the dissolution profile of poorly aqueous soluble drugs. In the present study, for bioavailability improvement of DRV, two thrust areas were emphasized, firstly increasing its solubility by means of SD and secondly inhibiting its P-gp mediated efflux by incorporating polymer with pharmacokinetic modulatory property [10]. Therefore, SD was opted as a suitable approach for enhancing solubilization of DRV. This formulation strategy of SD combats major concerns related to the other methodologies such as physical stability of the drug, since in many cases the amorphous form readily recrystallizes into the more stable crystalline form losing the advantage of increased dissolution rate and increased solubility and finally compromising the bioavailability of such drugs [11].

Second approach to increase the systemic availability of DRV is to hamper the drug efflux through P-gp. Coadministration of P-gp inhibitors (therapeutic agents) would result in increase in bioavailability but the toxicity associated with their high dose (required for P-gp inhibition) limits their usage. Ritonavir is the most widely used therapeutic agent for the inhibition of P-gp efflux pumps, thus contributing as a pharmacokinetic booster when given with antiretroviral therapies [12]. Pharmaceutical excipients, which are largely used as inert vehicles in drug formulations with no pharmacological actions of its own, are emerging as a special class of P-gp inhibitors [13]. Pharmaceutical surfactants which have already been approved for use in pharmaceutical formulations seem to be a better choice since they interact directly with the lipid bilayer plasma membrane, inserting themselves in between them and thereafter fluidizing them. Some of these surfactants include C8/C10 Glycerol and PEG Esters, Sucrose Esters, Polysorbates, and Tocopherol Esters [12].

Simple carriers without surfactant properties have been used earlier in order to enhance bioavailability but the carriers with surfactant properties have not been investigated to a wide extent as they possess potential to achieve anticipated bioavailability. These carriers with surfactant like properties have given manifold improved bioavailability in comparison to simple carriers and hence can be preferred over them. Interestingly, utilizing the carriers which possess P-glycoprotein inhibitory activity could potentially enhance the bioavailability of substrate drugs and thus will further add to the therapeutic effect. Therefore, in the present work, Kolliphor as the carrier which exhibits surfactant and P-gp inhibitory activity has been used in order to enhance solubility resulting in enhancement of related bioavailability of an anti-HIV drug, Darunavir (DRV). However, there is no previous study reporting the preparation of solid dispersion of DRV. Some formulation approaches attempted in the past aiming to increase the bioavailability of DRV were complexation of DRV to *β*-cyclodextrin [14] and production of pellets of DRV using wet extrusion/spheronization with kappa-carrageenan or microcrystalline cellulose (MCC) as pelletization aid [15]. However, both the approaches were targeted to increase the solubility and bioavailability of DRV but did not focus on increasing the intestinal uptake by inhibition of P-gp transporters present on the intestinal epithelium.

In the present study, SDs of DRV were prepared by employing design of experiment (DoE) to investigate different methods of preparation (like melt, solvent evaporation, and spray drying) and to screen and optimize carriers which are surface-active agents and P-gp inhibitors. The effect of food on absorption of drug was also studied both* in vitro* (in biorelevant media) and* in vivo*. Therefore, the formulation prepared by using this strategy is hypothesized to increase the solubility of the poorly soluble drug DRV as well as inhibiting its efflux by P-gp which can lead to an improved* in vivo* prospect and increased therapeutic efficacy. To our knowledge, this is the first study reporting the formulation of SD of DRV using novel polymer Kolliphor TPGS exhibiting P-gp inhibiting potential.

Till date, third-generation carrier has not been used for the bioavailability enhancement of an anti-HIV agent. Furthermore, Darunavir is an anti-HIV agent which has not been extensively worked upon. Only few research papers are available whose rationale was entirely different from the rationale of the present work. Although the concept of using P-gp inhibitor has also been investigated by a large number of research groups, the use of third-generation carrier with P-gp inhibiting activity for the solubility and bioavailability enhancement of an anti-HIV agent has not been studied previously. Therefore, the present work is novel and remains critically unexplored so far.

## 2. Material and Method

### 2.1. Material

Darunavir Ethanolate, Ritonavir, and Atazanavir Sulphate were received as a gift sample from Ranbaxy Research Laboratory (Gurgaon, India). Soluplus, Kolliphor TPGS (d-alpha-tocopheryl polyethylene glycol 1000 succinate), and Poloxamer 188 were obtained from BASF (Ludwigshafen, Germany). PVP K30 (Polyvinylpyrrolidone K30) was obtained from ISP (New Jersey, USA) and HPMC E5 (hydroxypropyl methylcellulose) from Dow Chemical Company (Michigan, USA). Diethyl ether and ethanol were obtained from Merck (Mumbai, India). SIF (Simulated Intestinal Fluid) powder was from Phares AG (Basel, Switzerland). Other chemicals and reagents used were from SD Fine Chemicals, Ltd. (Mumbai, India), and Qualigens Fine Chemical (Mumbai, India) and were of analytical grade. All drug solutions and buffer solutions were freshly prepared before use.

### 2.2. Formulation Design and Development

#### 2.2.1. Selection of Carrier

From literature search, six different carriers possessing P-gp inhibiting activity like Poloxamer 188, Kolliphor TPGS, Tween 80, Soluplus, Povidone (PVP K30), and Hypromellose (HPMC E5) were selected and were screened out by phase solubility study to determine the most appropriate and suitable carrier. In phase solubility study, an excess amount of DRV (100 mg) was added to 10 mL of distilled water, each containing different concentrations of carriers (i.e., 0.5%, 1%, 1.5%, and 2% w/v). The samples were prepared in triplicate and shaken in an oscillating water bath (Metrex Scientific Instrument, New Delhi, India) thermostatically controlled at 37 ± 0.5°C for 72 h and then centrifuged (Remi R-244, Mumbai, India) at 4,000 rpm for 15 min. The samples were filtered through a 0.45 *μ*m membrane filter, suitably diluted, and analyzed spectrophotometrically by UV spectrophotometer UV -2450 (Shimadzu, Kyoto, Japan) at 264 nm [16].

#### 2.2.2. Custom Design

Soluplus and Kolliphor TPGS were selected based on the phase solubility study as a carrier. From the design of experiment (DoE), custom design was applied to screen (i) method of preparation of solid dispersion, (ii) polymer as a carrier, and (iii) drug to polymer ratio as input variables. The custom design studied the interaction of various categorical and continuous parameters and their effect on the dissolution rate of the drug and on cumulative percentage drug release. The polymer (Soluplus and Kolliphor TPGS) and the method of preparation (melt, solvent evaporation, and spray drying) were selected as the categorical factor and the drug : polymer ratio (1 : 0.5, 1 : 1.25, and 1 : 2) was selected as the continuous factor. The response factor (output variable) of the study was % cumulative drug release at 30 min and 60 min. A set of 18 experiments were generated by software (JMP software version 9) (Table 1). Experiments were performed in same randomization sequence as given by software.

#### 2.2.3. Preparation of Solid Dispersion

*Solid Dispersion by Melt Method.* Accurately weighed quantity of polymer (Soluplus or Kolliphor) as per experiment numbers 3, 9, 11, 14, 16, and 18 of DoE was taken in a china dish and melted over a hot plate (Scientific Equipment, New Delhi, India) at temperature above melting point of polymer (melting point of Soluplus 90 ± 2°C and of Kolliphor TPGS 60 ± 2°C). The drug was weighed and added to the molten mass of polymer and the mixture was stirred continuously for 10 min using a glass rod and then allowed to cool in an ice bath. The dispersion of drug prepared using Soluplus was solidified, scraped, pulverized, sieved with BSS (British Standard Sieve) sizes, sieve number 40, and stored in a desiccator. The dispersions obtained using Kolliphor TPGS were semisolid waxy substance which were scraped and stored in a desiccator.

*Solid Dispersion by Solvent Evaporation Method.* Accurately weighed quantity of polymer (Soluplus or Kolliphor) as per experiment numbers 1, 4, 6, 8, 10, and 12 of DoE was dissolved in ethanol in a beaker using an overhead stirrer (RQ 124A, Universal Motors Ltd., Mumbai, India). Accurately weighed amount of DRV was then added to the polymer solution with continuous stirring for around 45–60 min until a clear solution was obtained. The clear solution was poured in Petri-plates and dried at 40 ± 2°C in an oven under vacuum. The dried samples were scraped, pulverized, sieved with BSS sieve number 40, and kept in a desiccator. 

*Solid Dispersion by Spray Drying Method.* As per experiment numbers 2, 5, 7, 13, 15, and 17 of DoE, accurate quantity of Soluplus was dissolved in ethanol and Kolliphor TPGS was dissolved in dichloromethane (DCM) using an overhead stirrer. Accurately weighed amount of DRV was then added to the respective polymer solutions with continuous stirring for around 45–60 min until a clear solution was obtained. This final clear solution was used as a feeding solution and sprayed via a fluid nozzle from a Mini Spray Dryer (Buchi, Switzerland). Nitrogen gas was used as the drying gas at an atomization pressure of 4 bars with the aspirator set to 100% so that the oxygen concentration remains below 6%. The flow rate of the feed solution was set to 5 ml/min. After the completion of the spray drying step, the samples were transferred into a freeze dryer and freeze dried for 72 h. The powder finally obtained was further dried in a vacuum dryer for 24 h and then sieved through sieve BSS 40.

### 2.3. Evaluation of Solid Dispersion

#### 2.3.1. Saturation Solubility Study

Saturation solubility study was carried out to determine the increase in the solubility of pure drug in the prepared SDs. Excess amount of the prepared SDs was added to 10 ml of distilled water in glass vials. Samples were kept in triplicate on a water bath shaker at 37 ± 0.5°C for 48 h, after which they were filtered through 0.45 *μ*m PVDF (polyvinylidene fluoride) membrane filters (from Millipore), suitably diluted, and analyzed by UV spectrophotometer at 264 nm.

#### 2.3.2. Drug Content Determination

SDs equivalent to 80 mg of DRV were added to 250 ml of methanol contained in a volumetric flask. Samples were sonicated for 15 min and filtered using a 0.45 *μ*m PVDF filter. After suitable dilution, the drug content was determined using the calibration curve of pure drug in methanol by UV analysis.

#### 2.3.3. *In Vitro* Dissolution Study

Dissolution studies were performed using a USP II Paddle dissolution apparatus (Distek, USA). The prepared SDs were filled in 00 size capsules and placed in Japanese sinkers. The sinkers were then placed in dissolution vessels containing 900 ml of acetate buffer pH 4.5, at 75 rpm and temperature 37 ± 0.5°C. Samples (5 ml) were collected periodically at 15, 30, 45, 60, and 90 min, filtered through a 0.45 *μ*m PVDF filter and replaced with a fresh dissolution medium (5 ml). The concentrations were analyzed at 264 nm by UV spectrophotometer.

### 2.4. Physicochemical Characterization of Optimized Solid Dispersion

*Differential Scanning Calorimetry* (DSC) was performed for DRV, Kolliphor TPGS, and SD7 in Jade DSC (Perkin Elmer, Massachusetts, USA). The samples were heated in a temperature range of 30–300°C in pierced aluminium pans. The heating rate was set to 10°C/min. Inert atmosphere was maintained by purging nitrogen gas at a flow rate of 20 ml/min.

*Fourier Transform Infrared* (FTIR) analysis was carried out with the help of Bruker alpha-T spectrophotometer, Ettlingen, Germany. Samples equivalent to 5 mg were placed on the sample holder. The overhead compressor was put on the sampling point to allow transmittance to occur. The spectra were recorded by scanning the pellet between 4000 and 400 wavelengths (cm^−1^).

*X-Ray Diffraction* (XRD) analyses were performed using an X-ray diffractometer Bruker D8 ADVANCE (Bruker, USA), equipped with CU-anode. Copper was the source of radiation operated at 30 kV, 40 mA, and a nickel filter was used to strip K beta radiation. The shift or change in the 2*θ* values was obtained.

*Scanning Electron Microscopy* (SEM) helped in determining surface morphology of DRV and its SD. Prior to examination, samples were mounted on an aluminium stub using a double sided adhesive tape and then making it electrically conductive by coating with a thin layer of platinum (approximately 20 nm) in vacuum. It was operated at an acceleration voltage of 1.9 kV.

### 2.5. Dissolution Studies in FaSSIF and FeSSIF Media

The dissolution profile of the SD7 was also performed in FaSSIF (fasted state simulated intestinal fluid) and FeSSIF (fed state simulated intestinal fluid) media to determine the effect of food on the absorption of DRV in the intestinal tract. These two biorelevant media, FaSSIF and FeSSIF, were developed to simulate the condition of the intestine in the fasted and fed states [17].

#### 2.5.1. Preparation of FaSSIF Media

Blank FaSSIF was prepared by dissolving 6.186 g NaCl, 4.47 g NaH_2_PO_4_, and 0.348 g NaOH in 900 ml of distilled water and the pH was adjusted to 6.5 by using 1 N NaOH or HCl using pH meter. The volume was made up to 1000 ml using distilled water.

Blank FaSSIF medium (500 ml) was taken and 2.240 g SIF powder was added to it and this solution was magnetically stirred until the powder completely dissolved to obtain a clear micellar solution. The volume was made up to 1000 ml using the buffer. The solution was allowed to stand for 2 h; it becomes slightly opalescent and thereafter used. A volume of 500 ml is recommended for dissolution for simulating fasted state condition

#### 2.5.2. Preparation of FeSSIF Media

Blank FeSSIF was prepared by dissolving 8.65 g glacial acetic acid, 11.874 g NaCl, and 4.04 g NaOH in 900 ml of distilled water and the pH was adjusted to 5.0 by using 1 N NaOH or HCl using pH meter. The volume was made up to 1000 ml using distilled water.

Blank FeSSIF medium (500 ml) was taken and 11.20 g SIF powder was added to it and this solution was magnetically stirred until the powder completely dissolved to obtain a clear micellar solution. The volume was made up to 1000 ml using the buffer and thereafter used. For simulating fed state condition 1 L dissolution fluid is recommended.

### 2.6. *In Vitro* Permeation Study of the Optimized Solid Dispersion

*In vitro *permeation study of the optimized SD (SD7) was carried out with the help of everted gut sac method. The present study was aimed at investigating the effect of Kolliphor TPGS as P-gp inhibitor on the intestinal absorption of DRV. The experiments were performed on adult male albino Wistar rats (150–200 g) obtained from Central Animal House, Jamia Hamdard (Approval number 1075). They were housed in cages with a 12 h light/dark cycle with free access to water and maintained on the feed. After overnight fasting (10–12 h), the rats were anesthetized with diethyl ether and the small intestine at the ileocecal junction was isolated and rinsed with Tyrode buffer solution pH 7.4 (containing in mM: 15 glucose, 11.90 NaHCO_3_, 136.9 NaCl, 4.2 NaH_2_PO_4_, 2.7 KCl, 1.2 CaCl_2_, and 0.5 MgCl_2_) at room temperature. The intestinal segment was immediately transferred to oxygenated Tyrode solution (95% O_2_ and 5% CO_2_) maintained at 37 ± 0.5°C and was cleared off the adhering tissue and rinsed with Tyrode solution. The tissue was continuously aerated (oxygenated) with the aid of an electrical aerator. Intestinal segments of 4 cm were cut and ligated with nylon thread at one end and carefully everted on the glass rod. The everted gut sacs were filled with 500 *μ*l of Tyrode solution and ligated at the other end and then placed inside the test tube containing 15 mL of the test solution continuously bubbled with atmospheric air at 15–20 bubbles per min separately. Test tubes were maintained at temperature 37 ± 0.5°C on water bath. The test solution outside the sac was termed as mucosal fluid, and the solution inside the sac was termed as serosal fluid. The amount of drug permeated across the intestine in serosal fluid was determined using HPLC method after predetermined time intervals (15, 30, 45, 60, 90, and 120 min); HPLC with UV detector method was employed for quantification of DRV in SD formulation. The mobile phase used for quantification of DRV consisted of acetonitrile and water (40 : 60) at flow rate of 1 ml/min. The retention time (*R*_*t*_) was 4.192 min [18].

The experiment was conducted on three formulations including pure drug DRV (15 ml of 1 mg/ml solution), the conventional treatment, that is, DRV + Ritonavir (RTV) (15 ml of 1 mg/ml DRV + 1.7 ml of 1 mg/ml RTV calculated according to the dose of 8 mg/kg) and SD7 (15 ml equivalent to 1 mg/ml of DRV). Permeability coefficients (*P*_app_) of these formulations were calculated from mucosal to serosal direction according to the equation:

(1) $$\begin{array}{c} P_{app}\left(\;cm/sec\;\right)=\frac{\left(dQ/dt\right)}{\left(A*C_{o}\right)}, \end{array}$$

where the *dQ*/*dt* is the rate of drug permeation across the tissue, *A* is the cross-sectional area of the tissue, and *C*_*o*_ is the initial concentration in the donor compartment at *t* = 0 [19].

### 2.7. Pharmacokinetic Studies

The pharmacokinetic studies were performed to compare the plasma concentration profiles of the optimized SD with the pure drug and conventional therapy (DRV + RTV) to check the bioavailability difference between the two. The animal species used for* in vivo* experiments were adult albino Wistar male rats weighing 150–200 g obtained from Central Animal House, Jamia Hamdard. The research protocol of the animal experimentation was approved by Institutional Animal Ethics Committee (IAEC) of Hamdard University, New Delhi, India. The animals were housed four per cage at 20–24°C and 55 ± 5% relative humidity with free access to food and water with a 12-h light–dark cycle.

#### 2.7.1. Estimation of DRV in Plasma

A rapid, simple, specific, and accurate high performance liquid chromatography (HPLC) with UV detector method was employed for quantification of DRV in SD formulation in blood plasma as reported by Takahashi et al., 2007 [20]. The mobile phase used for quantification of DRV consisted of 39% 50 mM phosphate buffer (pH 5.9), 22% methanol, and 39% acetonitrile at flow rate of 1 ml/min. The retention time (*R*_*t*_), detection limit, and quantification limit were 3.458 min, 32.14 ng/ml, and 51.87 ng/ml, respectively. Internal standard used was Atazanavir. The accuracy and precision were excellent. The same method was employed for both fed and fasted conditions.

#### 2.7.2. Animals and Dosing Protocol

The albino Wistar rats were divided into two groups: one group contains the rats which were fasted overnight (fasted state), while the other group contains the rats which were fasted overnight and then fed for 15 min prior to dosing (fed state).

The dose was calculated according to the formula:

(2) Animal  Dose  (mg/kg)=HEDmg/kg×Km  factor  for  human  adultKm  factor  for  rat,

where HED is human equivalent dose in mg/kg, *K*_*m*_ factor for human adult equals 37, and *K*_*m*_ factor for rat equals 6

The fasted and fed groups were further subdivided into four groups with three animals in each group, one for control, the second for pure drug solution of DRV, third for the conventional treatment DRV + RTV, and fourth for the optimized preparation SD7. All the formulations were administered at the required dose (70 mg/kg of DRV, 8 mg/kg of Ritonavir) and were given orally using oral feeding sonde. The blood samples collected at appropriate time intervals were then analysed by HPLC and the pharmacokinetic parameters were estimated.

## 3. Results

### 3.1. Selection of Carrier

On the basis of the phase solubility study of DRV in different polymers, Soluplus and Kolliphor TPGS were selected as the two carriers for the formulation of SD based on the highest increase in the solubility of the drug in their respective aqueous solutions. The solubility of the drug in pure water was 0.15 mg/ml and in Soluplus and Kolliphor TPGS solution at drug : polymer ratio of 1 : 2 was 0.823 and 0.922 mg/ml, respectively. Thus, there was 5-6 times increase in the solubility of drug in water using these carriers* (the data is provided as Supplementary Material in Table S.1 available online at *https://doi.org/10.1155/2017/8274927*).*

### 3.2. Custom Design Analysis

The response variables generated for the runs of the custom design were analyzed to get the optimized formulation. The output variables (response variables) of the study were % cumulative drug release at 30 and 60 min. Regression plot for different output variables was obtained and the *P* value for both the regression plots was below 0.05 and *R*^2^ value was very close to 1 in both the cases. Based on this outcome, we concluded that the model is statistically relevant and could predict the response with minimum prediction error* (the data is provided as Supplementary Material in Figure S.1)*.

### 3.3. Optimization of Solid Dispersion

#### 3.3.1. Saturation Solubility Studies

The solubility profile of the prepared SDs was found to be directly proportional to the polymer concentration showing an increase with the increase of polymer concentration from 0.5 parts to 2 parts of the drug (Table 1). SD7 showed the highest saturation solubility of 1.36 mg/ml increasing the solubility of the drug by up to 9 times.

#### 3.3.2. Drug Content Determination

The results revealed that the drug content was found to be more in the SDs prepared using Kolliphor TPGS than with Soluplus. The highest % drug content was found to be 99.93 ± 0.341% for formulation SD7 (Table 1). The SDs prepared using Soluplus, on the other hand, exhibited a nonuniform pattern for the percent drug content, specifically those obtained by melt method.

#### 3.3.3. *In Vitro* Dissolution Study

The goal of the dissolution study was to illustrate the improvement in dissolution rate of various SDs over pure drug. Impact of polymer and method of SD preparation on drug release was studied through design of experiment. The output variables (response variables) of the design were % cumulative drug release at 30 and 60 min. The influence of individual parameters and their interaction terms on the response variables is known as Parameters Estimates.

Parameters for which Prob > |*t*| value was less than 0.05 had significant impact on response variable. For response of % drug release at 60 min, all individual terms (main effects) and 2nd-order interaction of polymer : drug ratio with polymer and method were significant terms and had significant impact on response variable* (the data is provided as Supplementary Material in Figures S.2 and S.3)*.

**Figure 1:**
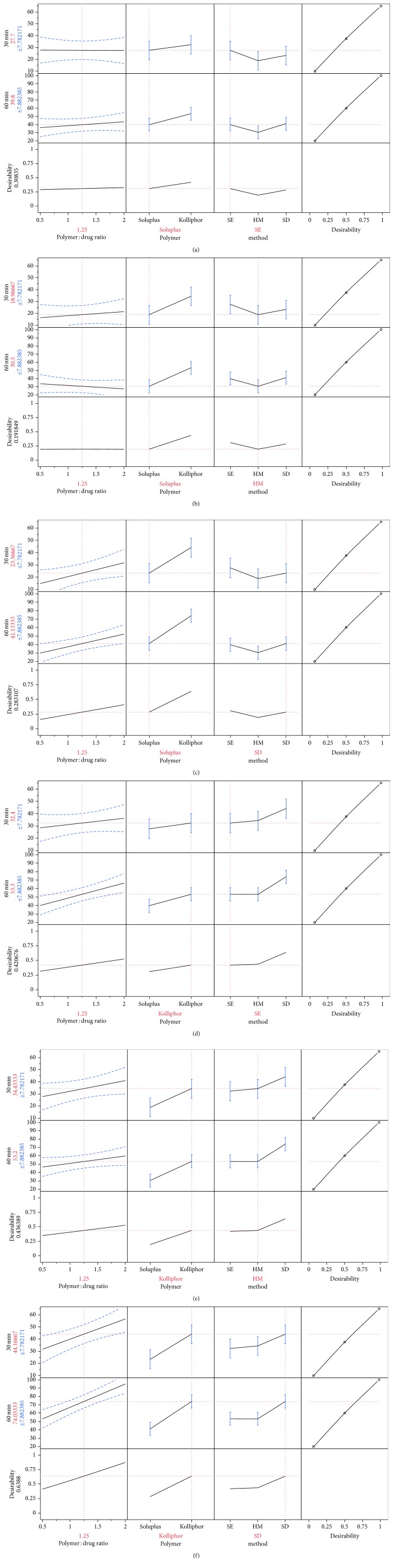
Prediction profiles generated by JMP software (version 9).

The prediction profiles were generated through the software during the custom design analysis as shown in Figure 1. Prediction profile showed graphical representation of correlation between input variables and output variables.

In Figures 1(a)–1(c), polymer Soluplus was selected and method was changed from solvent evaporation to spray drying to see the impact of different methods on response variables. The % drug release was found to increase with the increase in polymer : drug ratio. The polymer : drug ratio showed its impact on % drug release only in case of spray drying method, whereas for the melt and solvent evaporation method, negligible effect of polymer : drug ratio was seen. Thus, out of the three methods, spray drying was found to be the best method for solubility enhancement of drug. The desirability value obtained (0.283) was also the highest when spray drying method was used to make the solid dispersion.

In Figures 1(d)–1(f), Kolliphor TPGS was selected as the polymer and the method was changed to see the impact of different methods on response variables. In comparison to solvent evaporation and hot melt method, spray drying was found to be the best method for solubility enhancement of drug. The desirability value was also found to be highest, that is, 0.639, when choosing spray drying method. Line representing polymer : drug ratio was more inclined when spray drying method was selected for prediction of response variables. The polymer : drug ratio too exhibited its significant effect when spray drying method is selected for the preparation of solid dispersion.

Drug release was found to be the highest 98.8%, for the dispersion prepared by spray drying method using Kolliphor TPGS as the polymer in the drug : polymer ratio of 1 : 2 (SD7) which was almost 3 times of the drug release obtained with the drug alone i.e. 30.9%. However, the maximum drug release obtained by the dispersions using Soluplus as the polymer (SD2) by the same preparation method (spray drying) after 90 min was comparatively less i.e. 56.3% (Table 2). Hence the drug release was found to be dependent on the polymer used as well as the method of preparation.

### 3.4. Characterization of Optimized Solid Dispersion

**Figure 2:**
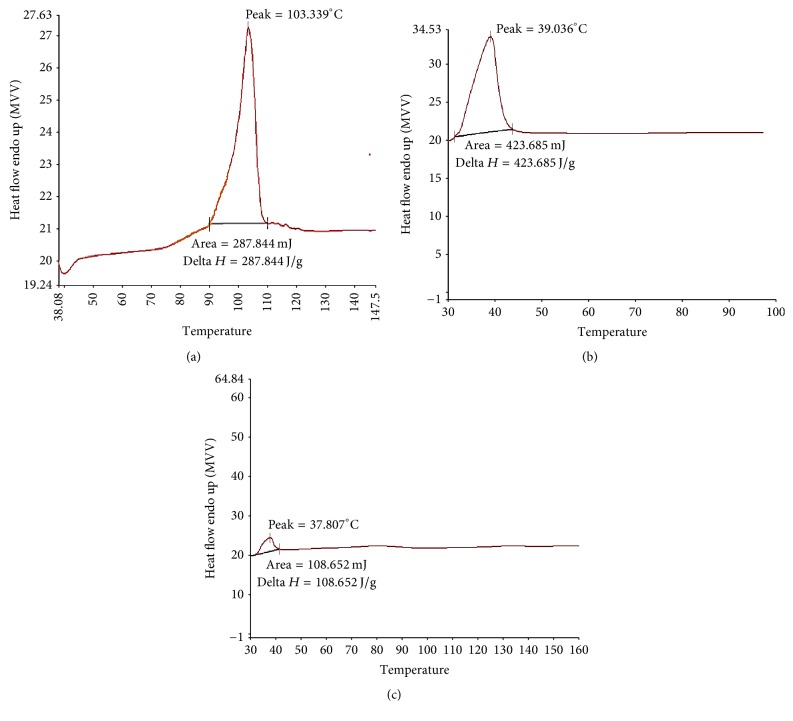
DSC of (a) DRV (b) Kolliphor TPGS and (c) SD7 = SD of DRV with Kolliphor TPGS.

The optimized formulation, that is, SD7 prepared using Kolliphor TPGS as the polymer in the drug : polymer ratio of 1 : 2 by spray drying method, was characterized by the following methods. DSC thermograms showed the melting endothermic peak for pure DRV at 103.3°C [Figure 2(a)] and at 39°C for Kolliphor TPGS [Figure 2(b)]. The thermogram of SD7 showed different thermal behaviour for DRV and peak characteristic to the polymer at 37.8°C was observed [Figure 2(c)].

The FTIR spectra of SD7 showed disappearance of peaks at 3448 and 1710 cm^−1^ which are characteristic of DRV. No sharp peak of DRV appeared in this region. However, there is a shift in the peak of polymer in the spectra and peaks at 2868 and 1093 cm^−1^ characteristic of Kolliphor TPGS are retained in the SD (*the data is provided as Supplementary Material in Figure S.4)*.

**Figure 3:**
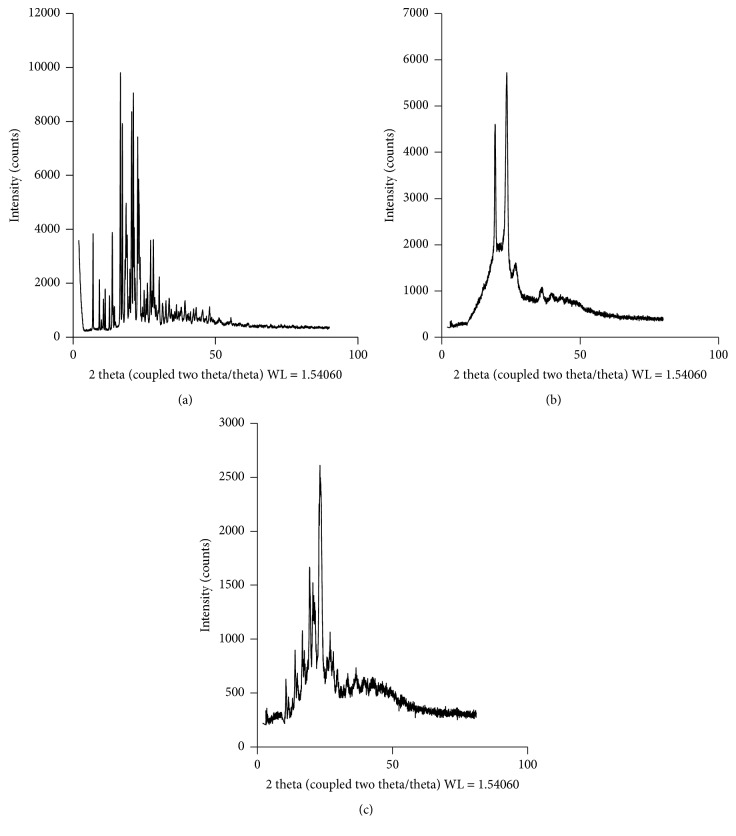
XRD of (a) DRV (b) Kolliphor TPGS and (c) SD7 = SD of DRV with Kolliphor TPGS.

Polymorphic transformation was observed with help of X-ray diffraction and results were found to be in good agreement with those of DSC. The crystalline nature of the drug was further confirmed by the appearance of sharp multiple peaks obtained in the XRD spectrum [Figure 3(a)], whereas these peaks were found to disappear in the case of spray dried dispersion confirming decrease in crystallinity [Figure 3(c)]. The observed few intensity peaks in the diffractograms are corresponding to those present in the polymer Kolliphor TPGS [Figure 3(b)].

**Figure 4:**
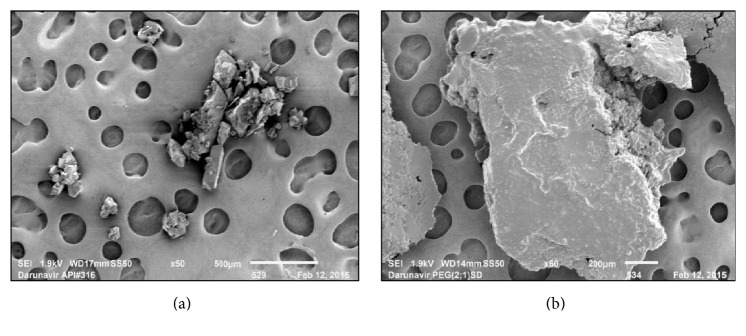
SEM images of (a) DRV and (b) SD7 = SD of DRV Kolliphor TPGS.

The SEM images indicated the morphology of the drug to be in crystalline rod shape [Figure 4(a)], whereas the smooth texture in SD depicts its amorphous character [Figure 4(b)]. It was also observed that the dimensions of the drug particles were much smaller in SD7.

### 3.5. Dissolution Studies in FaSSIF and FeSSIF Media

*In vitro* dissolution in biorelevant media (FaSSIF and FeSSIF) was performed for DRV and SD7 in order to observe the effect of food on the absorption of drug. The release of drug from SD7 increased from 72.6 ± 0.439 in FaSSIF to a maximum percent drug release of 99.6 ± 0.935 in FeSSIF. The extent of increase in the percentage drug release for the drug alone varied from 31.2 ± 0.425 in FaSSIF to 47.1 ± 0.534 in FeSSIF. The relative increase in the drug release in FaSSIF and FeSSIF media is depicted in Table 3.

### 3.6. *In Vitro* Permeation Study

*In vitro *permeation study by everted gut sac method was done to see the effect of P-gp inhibition characteristic of polymer on the absorption of the drug. The concentration of the drug inside the sac after 2 h was found to be 451.48 ± 4.02 *μ*g/ml and when given with Ritonavir, it was found to be 774.51 ± 2.81 *μ*g/ml. This increment in the drug concentration can be owed to the CYP3A4 as well as P-gp inhibiting activity of Ritonavir which enhances the absorption of the drug across the sac. The formulation SD7 showed the highest concentration of 2359.54 ± 2.95 *μ*g/ml exhibiting a fivefold increase in the drug concentration. Thus, Kolliphor TPGS proved to be a potential P-gp inhibitor in addition to its high solubilizing property.

*Apparent Permeability Coefficient.* The permeability coefficient (*P*_app_) was calculated and is represented in Table 4. The *P*_app_ of drug and drug in presence of Ritonavir was found to be 0.828 × 10^−6^ cm/sec and 1.389 ± 0.08 × 10^−6^, respectively, which is smaller than the permeability coefficient for SD7, that is, 5.899 ± 0.24  × 10^−6^ cm/sec by about 4 times.

### 3.7. Pharmacokinetic Studies

**Figure 5:**
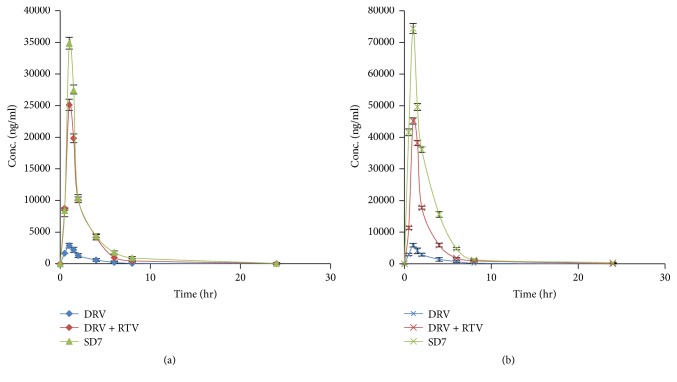
Plasma concentration profile of DRV following oral administration of different formulations in (a) fasted state and (b) fed state. Data presented as mean ± SD, *n* = 3 for each formulation.

The plasma conc. profile of DRV in albino Wistar rats following oral administration of SD7 formulation was compared with the plasma profile obtained following administration of pure drug and drug with RTV in fasting and fed state [Figure 5]. Plasma concentration-time profiles of DRV were evaluated by pharmacokinetic software (PK Functions for Microsoft Excel, Pharsight Corporation, Mountain View, CA). The pharmacokinetic profile clearly showed enhanced bioavailability of the formulations administered during the fed state as compared to the fasted state, thus indicating the significant effect of food on the bioavailability of the drug. The time (*T*_max_) to reach maximum plasma conc. (*C*_max_) was found to be the same in all the formulations which was 1 h. The *C*_max_ of formulation SD7, that is, 74441.59 ± 1598.09 *μ*g/ml (Table 5), is 10 times more than *C*_max_ value of drug (5874.93 ± 564.69 ng/ml) and almost double *C*_max_ value of DRV + RTV (45161.37 ± 979.13 ng/ml) in fed state. The high value of AUC (area under the curve) of SD7 in both fasting and fed state confirms greatly enhanced bioavailability of DRV.

Therefore,* in vivo* study findings demonstrated that Kolliphor TPGS incorporated in solid dispersion formulation of DRV exhibited tremendous potential for improving the solubilization and bioavailability of DRV.

## 4. Discussion

### 4.1. Selection of Carrier

On the basis of phase solubility studies, Soluplus and Kolliphor TPGS reported the maximum solubility of DRV. However, the solubility of DRV obtained with the carrier Kolliphor TPGS was observed to be slightly higher as compared to that with Soluplus. These results were in accordance with the study reported by Ramesh et al. wherein Kolliphor TPGS showed an enhanced solubility profile as compared to Soluplus in increasing the solubility of Etravirine [21].

### 4.2. Custom Design Analysis

The custom design is applied to the 18 formulations of DRV prepared with Soluplus and Kolliphor TPGS as the carrier to obtain the optimized formulation. This design helped in determining model's sensitivity to changes in the factor settings and to identify whether the model is fit for the experiment. The *P* value and *R*^2^ value further confirmed the fit of the custom design model for the experiment.

### 4.3. Optimization of Solid Dispersion

Kolliphor TPGS was selected as the carrier for preparing solid dispersion of DRV attributed to high saturation solubility, drug content, and % cumulative drug release. Further,* in vitro* permeation studies and* in vivo* studies have confirmed the P-gp inhibitory activity of Kolliphor TPGS which has resulted in its remarkable* in vivo* prospect.

#### 4.3.1. Saturation Solubility Studies

The increase in saturation solubility with increase in polymer concentration might be attributed to the physical properties of solubilizing and emulsifying ability of Kolliphor TPGS. It can also be due to possible complexation of the poorly soluble drug with water soluble carrier. Shin and Kim, 2003, reported a marked enhancement in the solubility from 18.25 to 345.75 *μ*g/mL of 1 : 2 (w/w) furosemide/TPGS solid dispersion, thus establishing a relevance to our results [22].

#### 4.3.2. Drug Content Determination

The high percent drug content for the SDs containing Kolliphor TPGS indicated uniform distribution of the drug in this hydrophilic carrier without any drug degradation and/or precipitation. Also, low values of standard deviation indicated the reproducibility of the method. Some variations were observed for the dispersions prepared using Soluplus, thus indicating improper distribution of the drug within the carrier. Similar results were obtained by Barea and coworkers who reported the drug content of thalidomide to be 117.2 ± 8.1% in the thalidomide : Kolliphor TPGS (1 : 4) solid dispersion owing to the uniform distribution of drug within the hydrophilic carrier [23].

#### 4.3.3. *In Vitro* Dissolution Study

On the basis of DoE study, it was seen in Figure 1 that all input variables exhibited a marked effect on the response variables. The response variables generated for the runs of the custom design were analyzed to get the optimized formulation along with the method of preparation. For SDs prepared using Kolliphor TPGS, polymer : drug ratio showed a remarkable increase in % drug release when spray drying method was used for formulation preparation. The reason for the high % of drug release with Kolliphor TPGS can be attributed to the improvement of wetting of drug particles due to surface-active property of polymer and localized solubilization by the hydrophilic nature of the polymer. Savjani et al. similarly optimized solid dispersion of itraconazole based on different polymers using DoE [24].

### 4.4. Characterization of Optimized Solid Dispersion

The optimized formulation, that is, SD7, on characterization by DSC showed disappearance of sharp melting peak of drug indicating a reduced degree of crystallinity and that the drug is either solubilized due to the presence of used excipients or present in an amorphous form. Fule and Amin observed similar results with solid dispersion of Lafutidine (LAFT) prepared by hot melt processing approach. The disappearance of peak of LAFT confirmed its conversion to its amorphous form [25].

Absence of peak characteristic of DRV and shift in the peaks of Kolliphor in FTIR spectra indicated presence of some interaction between drug and polymer. Bond formation between the drug and polymer might have occurred between drug and polymer. Similarly, Li et al. elucidated the reason behind disappearance of peaks of Curcumin in FTIR study to be as a result of interaction due to phenolic, carbonyl, and H-bond between Curcumin and Eudragit® E PO [26].

XRD revealed there might be transformation of crystalline DRV into amorphous form during the spray drying process as there was disappearance of peaks characteristic to DRV. The reason for this could be that spray drying is an energy intensive process where solution passes from the state of relative unsaturation to supersaturation in a fraction of seconds. Further, rapid evaporation of solvent from the supersaturated atomized droplets of the solution seemingly interferes with the crystal building process leading to amorphization of the drug. These results were in accordance with the results of Shamma and Basha who found out that the diffractogram of the solid dispersion of Carvedilol (CAR) showed complete absence of the distinctive peaks of CAR. The typical diffuse pattern obtained with the solid dispersion indicated the disruption of crystalline nature of CAR and changing into an entirely amorphous state [27].

SEM microphotographs revealed the presence of drug particles dispersed in the polymer matrix which was confirmed by the smooth texture exhibited in SD. There is no evidence of drug crystals, which confirms the previous findings based on XRD patterns. Similar results were obtained with the SEM results of solid dispersion of CoQ10 which confirmed the existence of CoQ10 in an amorphous form or very fine crystalline form [28].

### 4.5. Dissolution Studies in FaSSIF and FeSSIF Media

Increased release of DRV in FeSSIF confirms the higher concentration gradient of drug at the absorption site which will ultimately lead to an increased absorption. Furthermore, FeSSIF media facilitate this absorption by enhancing the solubilization of drug. So it was concluded that the formulation will be better absorbed when given with food. The study is in concordant with the study elucidated by Sinha and coworkers who demonstrated a better % drug release of solid dispersion of Ritonavir with Gelucire in FeSSIF media than FaSSIF indicating the effect of food on drug absorption [17]. Sekar et al. have also confirmed the increase in the absorption of DRV in the presence of food in a study done on healthy human volunteers [5].

### 4.6. *In Vitro* Permeation Study

The reason for the considerably lower value of *P*_app_ of DRV is owed to the presence of P-gp efflux transporters on the brush border membrane of intestinal epithelium which is responsible for attenuating the permeability of DRV. It also increases its level of exposure to cellular and luminal enzymes by effluxing the drug into the intestinal lumen or blood capillaries, thus metabolizing the drug [29]. The fourfold higher *P*_app_ value obtained with SD7 demonstrated that P-gp inhibition by Kolliphor TPGS could be considered as the most likely mechanism for enhancement of DRV absorption which allowed drug to permeate across the sac more easily and assisted in inhibiting the P-gp efflux of the drug. Thus, Kolliphor TPGS was proved to be a good choice as a polymer for enhancing the absorption of DRV. The potential of Kolliphor TPGS as a P-gp inhibitor was in accordance with that obtained by Varma and Panchagnula. Intrinsic permeability of paclitaxel in the presence of 1 mg/ml TPGS was found to increase from 0.08 × 10^−4^ to 0.22 × 10^−4^ cm/s. Thus, they demonstrated significant improvement in intestinal permeability of paclitaxel using Kolliphor TPGS [30].

### 4.7. Pharmacokinetic Studies

The study suggested that improved bioavailability in case of the SD of DRV in both fasted and fed states can be attributed primarily to the solubilizing activity and P-gp inhibiting action of Kolliphor TPGS incorporated in the dispersion. The carrier molecules fluidize the plasma membrane by inserting themselves between tails of the lipid bilayer, interacting with the bilayer's polar heads and thus modifying the hydrogen/ionic bond forces which may add onto their inhibitory action [31]. Also, the presence of food and moreover the possible size reduction might have occurred during the preparation which lead to easy availability of the drug and immediate drug release in the systemic circulation [32].

The* in vivo* data clearly indicated that Kolliphor TPGS can serve as a potential carrier for improving the solubility and bioavailability not only of orally administered Darunavir but also of other BCS class II–IV drugs which are P-gp substrates. The result was in concordance with that of the results obtained by Kim et al. wherein the effect of simultaneous administration of curcumin with P-gp substrate drug Saquinavir was studied. Curcumin owing to its P-gp inhibiting activity was found to increase the oral exposure of Saquinavir in rats by 2.7-fold [32].

## 5. Conclusion

Substantial solubility enhancement as well as P-gp inhibition was achieved for the drug DRV by formulating it into solid dispersion using Kolliphor TPGS as a carrier possessing P-gp inhibiting activity. The industrially scalable method, that is, spray drying, was optimized for the preparation of SD using DoE. Findings of DSC, XRD, FTIR, NMR, and SEM confirmed that DRV was present in an amorphous state in the spray dried SD at a drug : polymer ratio of 1 : 2 (w/w). The saturated solubility of DRV was markedly enhanced. The dissolution performed in biorelevant media exhibited maximum dissolution with FeSSIF media than in FaSSIF which confirmed food-related absorption of drugs. The P-gp inhibiting activity of the Kolliphor TPGS polymer was confirmed by both* in vitro* permeation studies and* in vivo* studies. The effect of food on absorption of drugs was also confirmed by conducting the pharmacokinetic studies in animals in both fasted and fed state. Thus, solid dispersions prepared using Kolliphor TPGS as a polymeric carrier and P-gp inhibitor together with spray drying as the preparation method represent a promising approach for enhancing the solubility, reducing the P-gp efflux, and finally improving the therapeutic efficacy of DRV.

## Supplementary Material

Table S.1: Mean solubility of drug in different polymers at three different ratios.
Figure S.1: Regression plot showing correlation coefficient of the drug release at various time intervals.
Figure S.2: Parameters estimation of responses of drug release studies at 30 min.
Figure S.3: Parameters estimation for responses of drug release studies at 60 min.
Figure S.4: FTIR of (a) DRV (b) Kolliphor TPGS and (c) SD7=SD of DRV with KolliphorTPGS.

## Figures and Tables

**Table 1:** Saturation solubility and % drug content of eighteen batches of solid dispersion (as predicted by Simple Custom Design) using Soluplus and Kolliphor TPGS. Data presented as mean ± SD, *n* = 3 for each formulation (*n* = 3).

Exp.	Formulation code	Polymer	Polymer : drug	Method	Saturation solubility (mg/ml) mean ± SD	% drug content
1	SD1	P1	0.5 : 1	SE	0.38 ± 0.005	98.16 ± 0.906
2	SD2	P1	2 : 1	S.D	1.12 ± 0.012	96.95 ± 0.527
3	SD3	P2	0.5 : 1	MM	0.59 ± 0.009	88.58 ± 1.238
4	SD4	P2	0.5 : 1	SE	0.54 ± 0.012	91.65 ± 0.951
5	SD5	P1	0.5 : 1	S.D	0.59 ± 0.006	91.81 ± 0.638
6	SD6	P2	1.25 : 1	SE	0.84 ± 0.018	93.23 ± 0.422
7	**SD7**	P2	2 : 1	S.D	1.36 ± 0.025	99.93 ± 0.974
8	SD8	P1	2 : 1	SE	0.98 ± 0.013	89.42 ± 0.894
9	SD9	P1	2 : 1	MM	0.69 ± 0.044	74.07 ± 0.637
10	SD10	P2	2 : 1	SE	1.02 ± 0.014	93.69 ± 0.691
11	SD11	P2	1.25 : 1	MM	0.74 ± 0.007	90.44 ± 0.721
12	SD12	P1	1.25 : 1	SE	0.42 ± 0.014	88.39 ± 0.418
13	SD13	P2	1.25 : 1	S.D	1.18 ± 0.015	99.09 ± 0.536
14	SD14	P1	1.25 : 1	MM	0.59 ± 0.041	67.09 ± 0.851
15	SD15	P2	0.5 : 1	S.D	0.83 ± 0.009	97.14 ± 0.711
16	SD16	P2	2 : 1	MM	0.99 ± 0.024	92.21 ± 0.632
17	SD17	P1	1.25 : 1	S.D	0.95 ± 0.012	93.51 ± 0.847
18	SD18	P1	0.5 : 1	MM	0.36 ± 0.048	59.19 ± 0.939

P1 = Soluplus, P2 = Kolliphor TPGS, MM = melt method, SE = solvent evaporation, and SD = spray drying.

**Table 2:** Comparative dissolution profile of nine batches of SD prepared using polymer P1 (Soluplus) and P2 (Kolliphor TPGS) in acetate buffer pH 4.5. Data presented as mean ± SD, *n* = 3 for each formulation.

Formulation code	Polymer	Cumulative % drug release (±SD) (*n* = 3)		
		After 30 min	After 60 min	After 90 min
SD1	P1	25.9 ± 0.541	37.4 ± 1.003	38.6 ± 0.536
SD2	P1	28.6 ± 0.312	48.7 ± 0.978	56.3 ± 0.612
SD3	P2	27.8 ± 1.012	48.9 ± 0.346	58.5 ± 0.830
SD4	P2	25.3 ± 0.664	34.6 ± 0.843	41.1 ± 0.964
SD5	P1	15.5 ± 0.718	30.3 ± 0.516	42.8 ± 0.521
SD6	P2	41.8 ± 0.992	61.9 ± 0.982	68.6 ± 0.843
SD7	P2	61.2 ± 0.813	98.5 ± 0.945	98.8 ± 1.262
SD8	P1	28.5 ± 1.408	42.2 ± 0.397	43.6 ± 0.325
SD9	P1	25.1 ± 0.119	34.0 ± 0.863	39.1 ± 1.463
SD10	P2	30.1 ± 0.761	63.4 ± 0.214	71.4 ± 1.332
SD11	P2	35.4 ± 0.218	54.9 ± 0.343	63.6 ± 0.525
SD12	P1	28.7 ± 0.558	39.8 ± 0.436	41.7 ± 0.358
SD13	P2	38.8 ± 0.592	70.8 ± 0.951	85.5 ± 1.175
SD14	P1	12.6 ± 1.117	23.5 ± 1.265	37.3 ± 0.453
SD15	P2	32.5 ± 0.476	52.8 ± 1.478	63.3 ± 0.543
SD16	P2	40.1 ± 0.845	55.8 ± 0.659	64.6 ± 0.866
SD17	P1	26.0 ± 0.327	44.4 ± 1.367	55.5 ± 0.634
SD18	P1	19.2 ± 0.574	34.0 ± 0.512	39.1 ± 0.652

P1 = Soluplus; P2 = Kolliphor TPGS.

**Table 3:** Comparison between the cumulative % drug release in FaSSIF and FeSSIF media. Data presented as mean ± SD, *n* = 3 for each formulation.

Formulation code	Cumulative % drug release (±SD) (*n* = 3) in FaSSIF			Cumulative % drug release (±S.D.) (*n* = 3) in FeSSIF		
	After 30 min	After 60 min	After 90 min	After 30 min	After 60 min	After 90 min
Drug	13.8 ± 0.239	25.7 ± 0.512	31.2 ± 0.425	13.7 ± 0.362	36.7 ± 0.753	47.1 ± 0.534
SD7	42.0 ± 0.515	61.1 ± 0.626	72.6 ± 0.439	61.3 ± 0.944	88.2 ± 0.873	99.6 ± 0.935

SD7 = (DRV : Kolliphor TPGS = 1 : 2 w/w, spray drying).

**Table 4:** Apparent permeability coefficients *P*_app_ (cm/s). Data presented as mean ± SD, *n* = 3 for each formulation.

Formulation code	Equation of graph between drug conc. inside sac (*µ*g/ml) and time (sec)	*R* ^2^	Apparent permeability coefficient (×10^−6^ cm/sec) mean ± SD
Drug	0.0375*x* + 187.53	0.9901	0.828 ± 0.12
Drug + Ritonavir	0.0629*x* + 326.98	0.9959	1.389 ± 0.08
SD7	0.267*x* + 447.32	0.9995	5.899 ± 0.24

SD7 = (DRV : Kolliphor TPGS = 1 : 2 w/w, spray drying).

**Table 5:** Results for various pharmacokinetic parameters by different formulations in Wistar rats. Data presented as mean ± SD, *n* = 3 for each formulation.

Groups	Formulations	Pharmacokinetic parameters				
		*C*_max_ (ng/ml)	*T*_max_ (h)	AUC_0-*t*_ (ng·min/ml)	AUC_0-*α*_ (ng·min/ml)	*K*_el_
Group A (fasted)	DRV	2864.28 ± 365.83	1	6945.18 ± 117.63	7033.51 ± 119.94	0.211 ± 0.002
	DRV + RTV	25115.75 ± 895.96	1	52036.81 ± 406.93	52182.84 ± 397.10	0.266 ± 0.002
	SD7	34820.79 ± 991.21	1	67269.08 ± 407.25	67500.64 ± 436.42	0.252 ± 0.006

Group B (fed)	DAR	5874.93 ± 564.69	1	15451.98 ± 61.65	15556.26 ± 59.63	0.228 ± 0.003
	DRV + RTV	45161.37 ± 979.13	1	90985.44 ± 440.90	91232.40 ± 465.63	0.264 ± 0.008
	SD7	74441.59 ± 1598.09	1	172727.90 ± 919.17	174292.46 ± 880.76	0.225 ± 0.001

(*C*_max_ = peak plasma concentration; *T*_max_ = time taken to reach *C*_max_; AUC_0-*t*_ = area under curve from 0-*t*; AUC_0-*α*_ = area under curve from 0-*α*; *K*_el_ = elimination rate constant).
