# Supplementary material for: Role of P-Glycoprotein Inhibitors in the Bioavailability Enhancement of Solid Dispersion of Darunavir
Source: Biomed Res Int. 2017 Oct 31;2017:8274927. doi: 10.1155/2017/8274927 (PMC5684613; doi:10.1155/2017/8274927)
Supplement: Supplementary file 1 — Table S.1: Mean solubility of drug in different polymers at three different ratios. Figure S.1: Regression plot showing correlation coefficient of the drug release at various time intervals. Figure S.2: Parameters estimation of responses of drug release studies at 30 min. Figure S.3: Parameters estimation for responses of drug release studies at 60 min. Figure S.4: FTIR of (a) DRV (b) Kolliphor TPGS and (c) SD7=SD of DRV with KolliphorTPGS. [file 8274927.f1.pdf]

SUPPLEMENTARY MATERIAL

Supplementary Material Legends

Table S.1: Mean solubility of drug in different polymers at three different ratios

Figure S.1: Regression plot showing correlation coefficient of the drug release at various time intervals

Figure S.2: Parameters estimation of responses of drug release studies at 30 min.

Figure S.3: Parameters estimation for responses of drug release studies at 60 min.

Figure S.4: FTIR of (a) DRV (b) Kolliphor TPGS and (c) SD7=SD of DRV with KolliphorTPGS

Table S.1: Mean solubility of drug in different polymers at three different ratios

Polymer	Mean solubility ± S.D. (mg/ml) (n=3)			
	**Drug:Polymer (1:0.5)**	**Drug:Polymer (1:1)**	**Drug:Polymer (1:1.5)**	**Drug:Polymer (1:2)**
Soluplus	0.346 ± 0.017	0.535 ± 0.016	0.654 ± 0.019	0.823 ± 0.023
Kolliphor TPGS	0.427 ± 0.016	0.622 ± 0.024	0.681 ± 0.020	0.922 ± 0.025
Tween 80	0.270 ± 0.019	0.397± 0.019	0.595 ± 0.016	0.720 ±0.020
PVP-K30	0.168 ± 0.018	0.232 ± 0.016	0.243 ± 0.016	0.316 ± 0.019
HPMC E5	0.167 ± 0.007	0.198 ± 0.013	0.249 ± 0.018	0.243 ± 0.019
Poloxamer 188	0.216 ± 0.014	0.281 ± 0.015	0.397 ± 0.013	0.400 ± 0.012

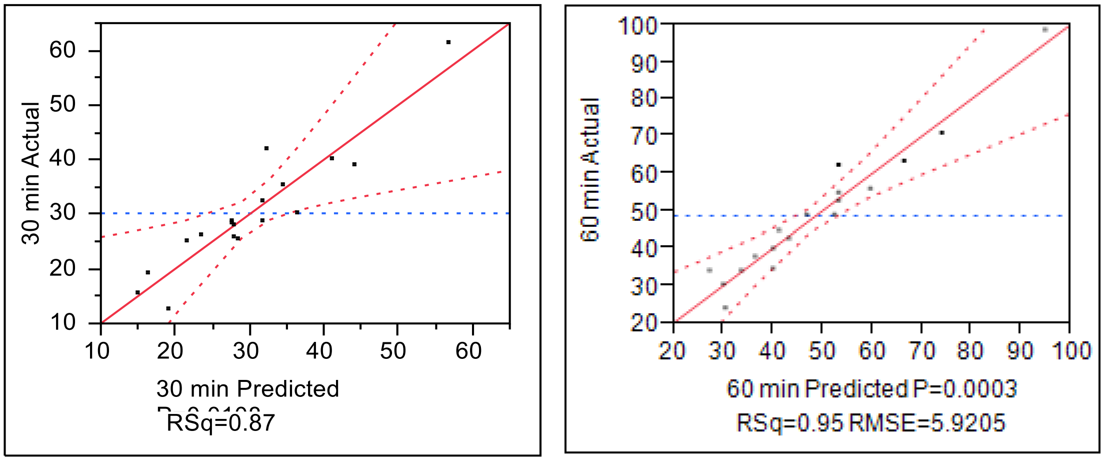

Figure S.1: Regression plot showing correlation coefficient of the drug release at various time intervals

Term	Scaled Estimate	Std Error	t Ratio	Prob>|t|
Intercept	30.172222	1.377733	21.90	<.0001*
Polymer : Drug ratio(0.5,2)	5.6166667	1.687371	3.33	0.0104*
Polymer[Soluplus]	-6.827778	1.377733	-4.96	0.0011*
Polymer[Kolliphor]	6.8277778	1.377733	4.96	0.0011*
method[SE]	-0.122222	1.948409	-0.06	0.9515
method[HM]	-3.472222	1.948409	-1.78	0.1126
method[SD]	3.5944444	1.948409	1.84	0.1023
Polymer : Drug ratio*Polymer[Soluplus]	-2.016667	1.687371	-1.20	0.2663
Polymer : Drug ratio*Polymer[Kolliphor]	2.0166667	1.687371	1.20	0.2663
Polymer : Drug ratio*method[SE]	-3.766667	2.386304	-1.58	0.1531
Polymer : Drug ratio*method[HM]	-1.066667	2.386304	-0.45	0.6667
Polymer : Drug ratio*method[SD]	4.8333333	2.386304	2.03	0.0774
Polymer[Soluplus]*method[SE]	4.4777778	1.948409	2.30	0.0506
Polymer[Soluplus]*method[HM]	-0.905556	1.948409	-0.46	0.6545
Polymer[Soluplus]*method[SD]	-3.572222	1.948409	-1.83	0.1041
Polymer[Kolliphor]*method[SE]	-4.477778	1.948409	-2.30	0.0506
Polymer[Kolliphor]*method[HM]	0.9055556	1.948409	0.46	0.6545
Polymer[Kolliphor]*method[SD]	3.5722222	1.948409	1.83	0.1041

Figure S.2: Parameters estimation of responses of drug release studies at 30 min.

Term	Scaled Estimate	Std Error	t Ratio	Prob>|t|
Intercept	48.661111	1.395475	34.87	<.0001*
Polymer : Drug ratio(0.5,2)	8.7166667	1.7091	5.10	0.0009*
Polymer[Soluplus]	-11.51667	1.395475	-8.25	<.0001*
Polymer[Kolliphor]	11.516667	1.395475	8.25	<.0001*
method[SE]	-2.111111	1.973499	-1.07	0.3160
method[HM]	-6.811111	1.973499	-3.45	0.0087*
method[SD]	8.9222222	1.973499	4.52	0.0019*
Polymer : Drug ratio*Polymer[Soluplus]	-4.85	1.7091	-2.84	0.0219*
Polymer : Drug ratio*Polymer[Kolliphor]	4.85	1.7091	2.84	0.0219*
Polymer : Drug ratio*method[SE]	-0.316667	2.417033	-0.13	0.8990
Polymer : Drug ratio*method[HM]	-6.991667	2.417033	-2.89	0.0201*
Polymer : Drug ratio*method[SD]	7.3083333	2.417033	3.02	0.0165*
Polymer[Soluplus]*method[SE]	4.7666667	1.973499	2.42	0.0422*
Polymer[Soluplus]*method[HM]	0.1666667	1.973499	0.08	0.9348
Polymer[Soluplus]*method[SD]	-4.933333	1.973499	-2.50	0.0370*
Polymer[Kolliphor]*method[SE]	-4.766667	1.973499	-2.42	0.0422*
Polymer[Kolliphor]*method[HM]	-0.166667	1.973499	-0.08	0.9348
Polymer[Kolliphor]*method[SD]	4.9333333	1.973499	2.50	0.0370*

Figure S.3: Parameters estimation for responses of drug release studies at 60 min.

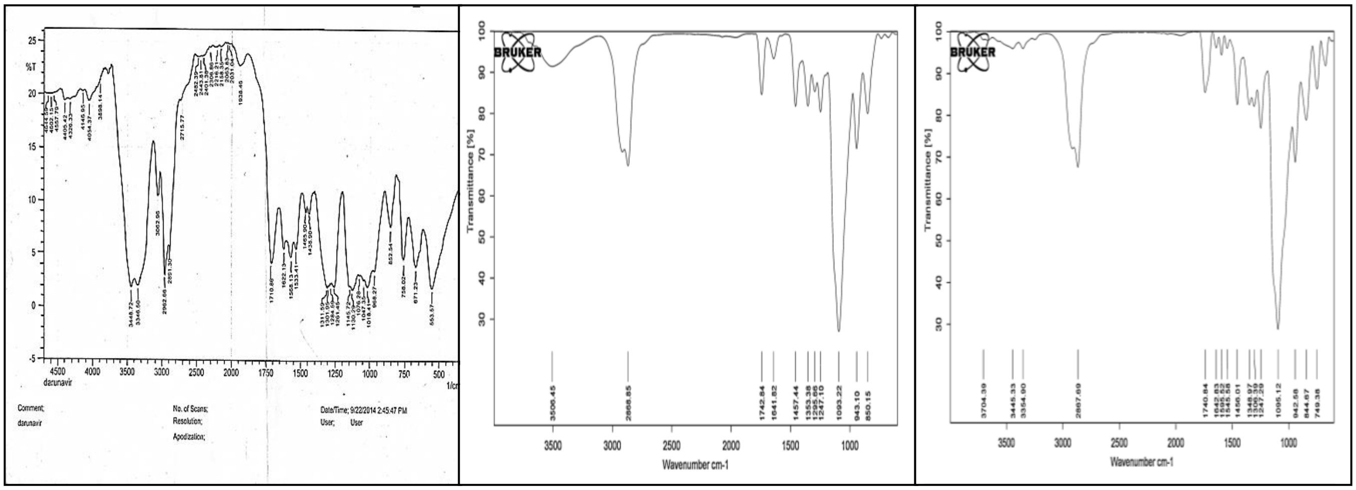

(a) (b) (c)

Figure S.4: FTIR of (a) DRV (b) Kolliphor TPGS and (c) SD7=SD of DRV with Kolliphor TPGS
